# A rare giant congenital intramural uterine cyst: a case report

**DOI:** 10.3389/fonc.2025.1691066

**Published:** 2025-12-01

**Authors:** Ping Su, Ben Wang, Su-wei Liu, Yi-ping Yuan, Jian Han

**Affiliations:** Department of Obstetrics and Gynecology, Daping Hospital, Army Medical University, Chongqing, China

**Keywords:** Müllerian remnants, mesonephric differential, intramural uterine cyst, ultrasound, pathology

## Abstract

Congenital intramural uterine cysts are extremely rare and often difficult to diagnose preoperatively. They are typically associated with remnants of the mesonephric or paramesonephric ducts and are pathologically benign; however, a very small number also carry a risk of malignancy. Imaging examinations can well aid in the diagnosis of uterine cysts, but because of their clinical rarity, the rate of misdiagnosis is very high. We report a case of a large congenital intramural uterine cyst. The preoperative ultrasound images showed a huge cyst in the pelvic area. The intraoperative images demonstrated the typical features of this case specimen: a cystic mass approximately 14 cm in diameter protruded from the left posterior wall of the uterus to the serous layer. Finally, we performed surgical resection of this cyst, and the pathology confirmed the diagnosis. Surgical treatment is the standard method for such cases.

## Introduction

Congenital intramural uterine cysts typically originate from embryonic remnants of the mesonephric or paramesonephric ducts. Because of insufficient understanding of these cysts, they are easily misdiagnosed as cystic adenomyosis or cystic degeneration of uterine fibroids; large ones are also frequently misdiagnosed as ovarian tumors. Although Sherrick and Vega proposed pathological diagnostic criteria for this disease as early as 1962 (1), a unified diagnostic standard and management protocol are still lacking due to its extreme rarity. Additionally, a very small number of paramesonephric duct-derived lesions carry a risk of malignancy (2). Clinical management requires individualized treatment based on factors such as the patient’s age and fertility requirements, with options including laparoscopic or transabdominal uterine cystectomy or hysterectomy. The disease is often identified during surgical treatment and confirmed by postoperative pathology. Given that congenital uterine cysts are extremely rare in clinical practice, their clinical diagnostic rate is low, and treatment is often delayed.

This case report describes the diagnosis and treatment of a large congenital uterine cyst in a middle-aged female patient, with unique characteristics in her symptoms and ultrasound reports. Our aim is to provide insights into the diagnosis and treatment of congenital uterine cysts.

## Case presentation

**Figure 1:**
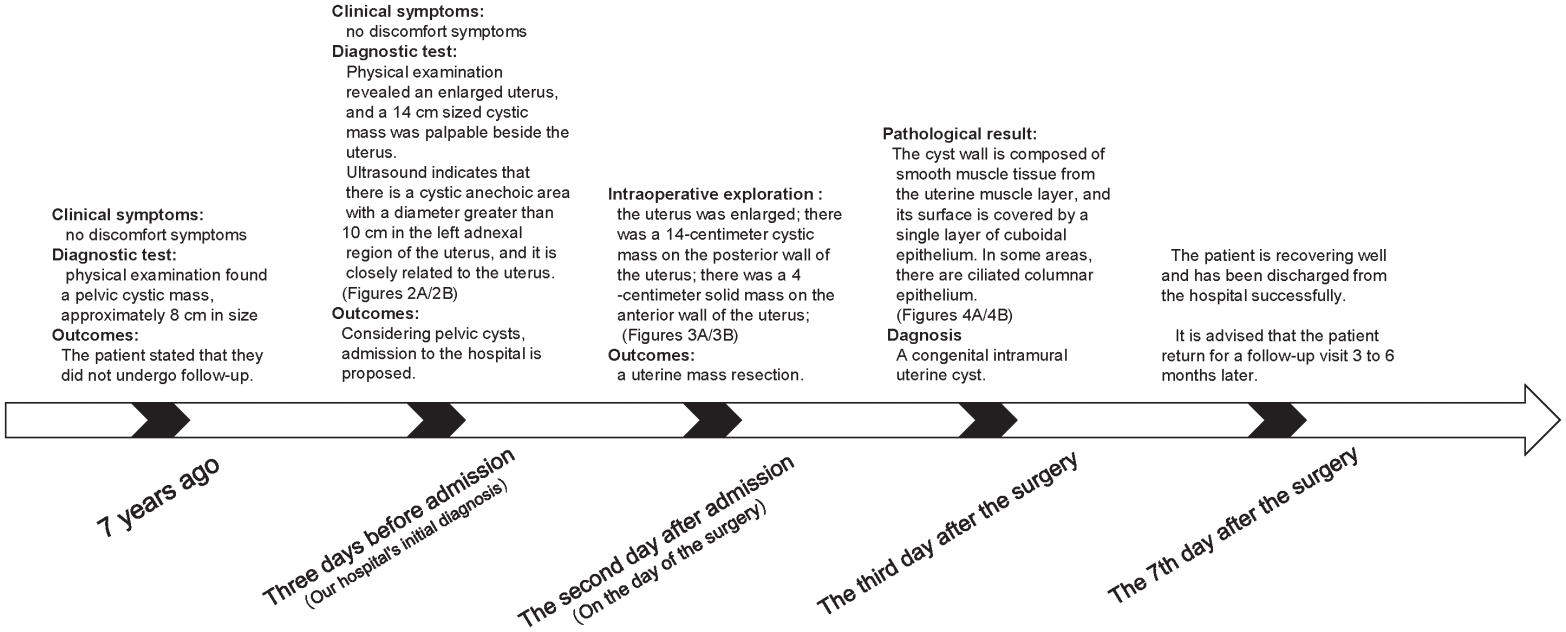
Timeline of the disease course.

**Figure 2:**
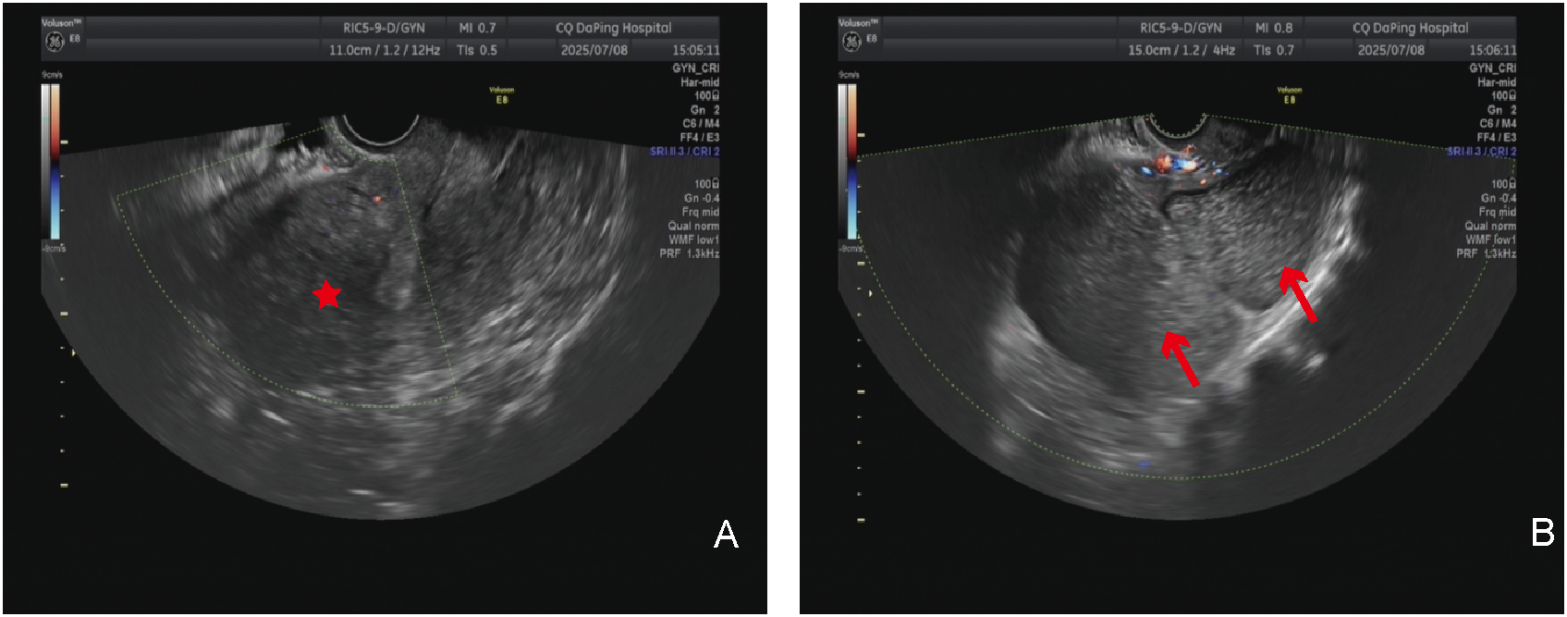
Imaging examination: **(A)** Transvaginal ultrasound examination; the red asterisk indicates the uterus. **(B)** Transvaginal ultrasound examination; the red arrow shows the giant cyst of the uterus.

The patient is a 51-year-old woman with regular menstruation. She reported that a pelvic cystic mass, approximately 8 cm in size, was detected during a physical examination 7 years ago. Previous medical reports from prior visits are missing, with specific details unknown, and she did not receive regular follow-up visits (**Figure 1**). She came to our hospital for a physical examination 3 days ago; on examination, the uterus was enlarged to the size of an 8-week pregnancy, and a 14-cm cystic mass was palpable adjacent to the uterus, with good mobility, close relation to the uterus, and no tenderness or discomfort. Gynecological ultrasound in the patient showed the following findings: (1) The anterior myometrium of the uterus presented with coarsened and enhanced echoes, with a heterogeneous echo area measuring approximately 4.9 × 4.8 cm, featuring ill-defined borders and no capsule noted. (2) A 14.1 × 9.7 cm cystic anechoic lesion was observed in the left adnexal region of the uterus, which was thick-walled with poor sound transmission and showed deposition of medium-echoic spots. This anechoic space-occupying lesion was closely related to the uterus. Ultrasound-based diagnostic considerations were as follows: (1) uterine adenomyoma and (2) cystic space-occupying lesion in the left adnexal region (O-RADS category: 3) (**Figures 2A, B**). Routine auxiliary examinations showed no abnormalities. Since the onset of the condition, the patient has had no discomforts such as increased menstrual flow, abdominal pain, abdominal distension, frequent urination, or constipation.

In summary of the above analysis, the attending physician admitted the patient to the hospital for management considering a pelvic cyst. A laparotomy was subsequently performed. Intraoperative exploration revealed the following: the uterus was enlarged to the size of a 2-month pregnancy; a cystic mass approximately 14 cm in diameter protruded toward the serosa from the left side of the posterior uterine wall, with approximately 90% protruding outside the serosa and soft in texture; a solid mass approximately 4 cm in diameter was present in the subserosal region of the anterior uterine wall, with approximately 80% protruding outside the serosa; and the bilateral fallopian tubes and ovaries showed no obvious abnormalities in appearance. The patient underwent uterine mass resection during the operation.

**Figure 3:**
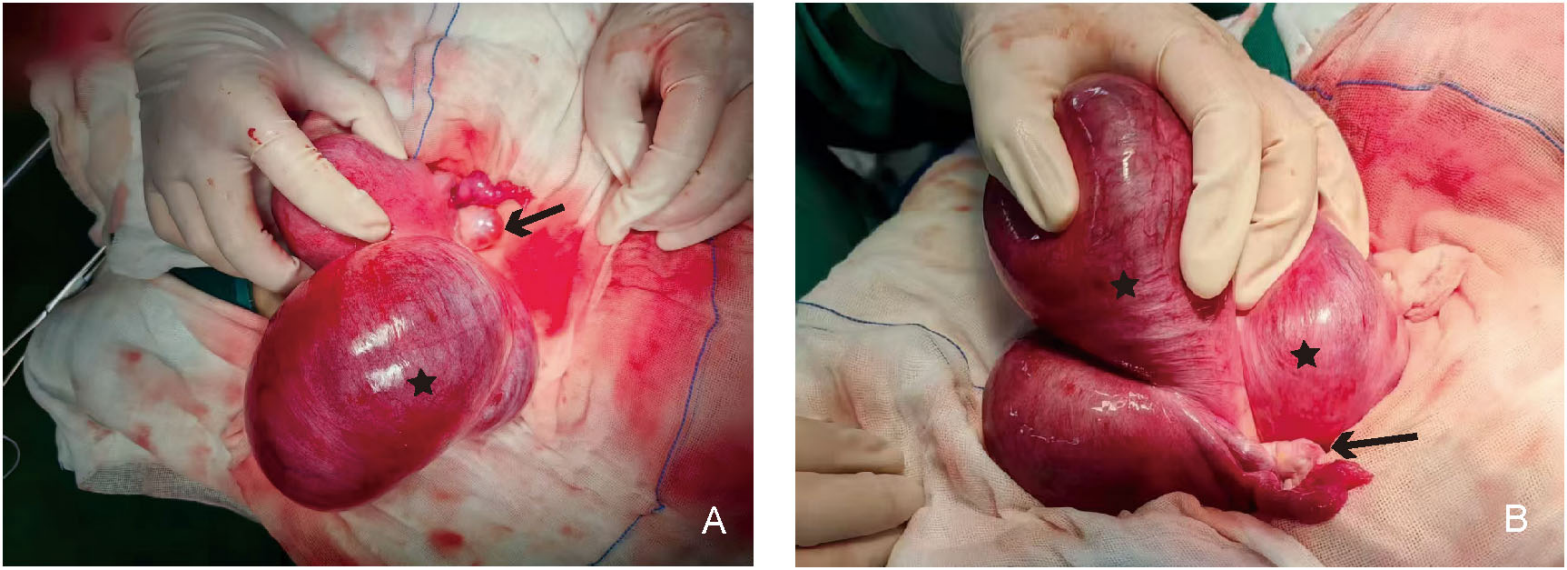
Gross specimen showing the relationship between the uterine cyst and the uterus. **(A)** Structural view of the anterior side of the uterine cyst: the black asterisk indicates the uterine cyst on the left side of the uterus, and the black arrow indicates the left ovary and fallopian tube. **(B)** Structural view of the posterior side of the uterine cyst: the black asterisk indicates the uterine cyst on the left side of the uterus, and the black arrow indicates the left ovary and fallopian tube.

**Figure 4:**
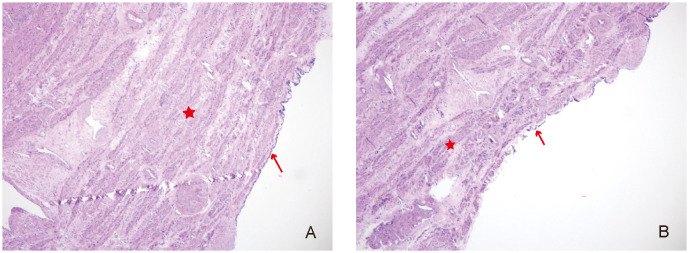
**(A, B)** Pathological examination of the uterus cyst (“H&E,×40”). The red asterisk shows the smooth muscle tissue, and the red arrow indicates cuboidal or columnar epithelium.

Gross examination of the specimen showed the following: the cyst was located below the left round ligament (**Figures 3A, B**), soft in texture, grayish-white, and thick-walled; pale yellow clear fluid was visible inside, and the inner wall of the cyst was smooth without papillary excrescences; and the anterior uterine wall had a solid, grayish-white, firm mass with a whorled structure inside. The operation was performed smoothly, and the patient was safely returned to the ward. Postoperative pathology of the patient indicated that the cyst wall was composed of smooth muscle tissue from the uterine myometrium, lined with a single layer of cuboidal epithelium, with a single layer of ciliated columnar epithelium lining some areas, leading to the diagnosis of a congenital uterine cyst (**Figures 4A, B**). The patient recovered well and was advised to attend a follow-up visit within 3–6 months.

## Discussion

Congenital intramural uterine cysts are extremely rare in humans, and all relevant literature consists of case reports (3). Congenital intramural uterine cysts exhibit high heterogeneity, with significant variations in size.

Uterus cysts can be categorized into two types: congenital and pathological. The latter includes cystic degeneration of myoma (4), adenomyotic cysts of the uterus (5), and cervical retention cysts (6), among others. Congenital intramural uterine cysts are not caused by the aforementioned pathological factors; instead, they are relatively rare cysts formed by residues of undegenerated mesonephric ducts and paramesonephric ducts during the development of the female reproductive system (7). During the embryonic stage, Müllerian ducts give rise to the female reproductive tract, and abnormalities in their development may lead to cyst formation. The congenital intramural uterine cyst may present as a unilocular cystic lesion within the uterine myometrium. Its anatomical location and morphological characteristics support the possibility that our case originates from the Müllerian ducts.

The tissue origin of congenital uterine cysts is closely related to their anatomical location. Cysts derived from the mesonephric duct primarily occur in the uterine cervix and can also be found in other anatomical sites where mesonephric duct remnants exist, mostly located in the lateral walls of the uterus. This distribution may be associated with the anatomical pathway of mesonephric duct remnants during the embryonic stage (8). For cysts of paramesonephric duct (Müllerian duct) origin, these lesions are typically situated in the lateral wall of the uterus near the round ligament and have no communication with the normal uterine cavity, which supports the theory of their paramesonephric duct origin (9).

On ultrasound, uterus cysts appear as hypoechoic or anechoic cystic masses (10). On pelvic computed tomography (CT), they present as thin-walled cystic masses, with cystic fluid predominantly showing low density (11). On pelvic magnetic resonance imaging (MRI), the cyst wall exhibits isointense signals on both T1-weighted (T1WI) and T2-weighted (T2WI) sequences; the cystic fluid shows high signal intensity on T1WI, while its signal intensity on T2WI is variable, which can be hypointense, isointense, or hyperintense. Uterus cysts may be associated with urogenital developmental anomalies (12). Therefore, routine corresponding imaging examinations are required preoperatively to clarify such conditions. The initial ultrasound examination did not take into account the cysts originating from the uterus. It was misdiagnosed as coming from the ovaries and was classified as O-RADS 3 level. We did not conduct CT/MRI examinations.

Congenital intramural uterine cysts are easily misdiagnosed as ovarian cysts on ultrasound. With the aid of CT and MRI, differential diagnosis still needs to be made from various uterine cystic diseases, including cystic degeneration of uterine fibroids, cystic adenomyosis, peritoneal encapsulated cysts, uterine mesothelial cysts, and juvenile cystic adenomyoma. Among these, the first two are the main diseases requiring differential diagnosis. The first is cystic degeneration of uterine fibroids, which arises from cystic changes due to necrosis and liquefaction of myocytes in uterine fibroids. It mostly has no obvious clinical manifestations, though a small number of cases complicated with infection may present with fever and lower abdominal pain (13). The second is cystic adenomyosis, which refers to cystic cavities (with a diameter of ≥5 mm) filled with ectopic endometrial tissue and bloody fluid, formed when ectopic endometrial tissue invades the uterine myometrium. Clinical manifestations may include dysmenorrhea, menorrhagia, and chronic pelvic pain. CA125 may be elevated; pathologically, the cyst wall epithelium is composed of endometrial glands and stroma, surrounded by smooth muscle tissue (14). In this case, ultrasound showed a cyst in the left adnexal region of the uterus with deposition of medium-echoic spots, which could also be easily misdiagnosed as cystic adenomyosis. However, the patient had no obvious dysmenorrhea, and intraoperatively, clear fluid was found—markedly different from the findings in cystic adenomyosis. This was further confirmed by postoperative pathology. In addition, peritoneal inclusion cysts are mostly associated with acquired pathological processes. The patient had previous discomfort, and during the surgery, the cyst was found to be located in the uterine muscle layer. It was clearly ruled out. Mesothelial cysts originate from the mesothelium of body cavities and are mostly related to the mesothelial tumor spectrum, which does not match the pathology of this case. Juvenile cystic adenomyoma is a specific cystic lesion associated with severe dysmenorrhea and has clear clinical and pathological features, which are significantly different from the symptoms and signs of this case.

Congenital intramural uterine cysts are generally benign lesions, with only a very small number of paramesonephric duct-derived lesions carrying a risk of malignancy. Clinical management requires individualized treatment based on factors such as the patient’s age, fertility requirements, and comorbidities; options include laparoscopic or transabdominal uterine cystectomy or hysterectomy. Pathological diagnosis adopts the diagnostic criteria proposed by Sherrick and Vega in 1962 (1): (1) The cyst does not communicate with the uterine cavity, and the cyst-lining epithelium is not endometrial epithelium. (2) The cyst is not connected to cervical endometrial glands, and the cyst-lining epithelium is not cervical endometrial epithelium. (3) The cyst is located within the uterine myometrium near the midline or at the lateral wall. (4) The inner wall of the cyst is lined with cuboidal or columnar epithelium, which may be ciliated or non-ciliated, or it resembles paramesonephric duct epithelium showing a low papillary pattern, or resembles mesonephric duct epithelium showing a flat pattern. (5) The cyst wall should contain a portion of the uterine myometrium. Surgical resection is the main treatment modality. If the specimens under microscopy are atypical, pathology needs to be combined with immunohistochemistry to confirm the nature. For example, juvenile cystic adenomyosis requires postoperative pathology to rule out malignancy (14).

## Conclusions

We report a case of a rare congenital intramural uterine cyst. The patient had a pelvic mass detected for over 7 years without abnormal symptoms or signs. Gynecological examination revealed a large palpable cystic mass in the left adnexal region. The primary preoperative diagnosis was ovarian cyst, but intraoperative findings revealed a large cystic uterine mass, and postoperative pathological diagnosis confirmed it as a congenital uterine cyst. Congenital intramural uterine cysts are extremely rare and often difficult to diagnose preoperatively. They usually originate from embryonic remnants of the mesonephric or paramesonephric ducts, mostly presenting as intramural cysts with clear fluid lined by a single layer of epithelium. Pathologically benign, their onset age is not particularly young due to insidious symptoms, and they do not appear to affect fertility. After intraoperative diagnosis, local cyst resection can achieve the therapeutic goal, and recurrence is extremely rare based on literature review. Immunohistochemistry is usually an important auxiliary method for pathological diagnosis.

## Data Availability

The original contributions presented in the study are included in the article/supplementary material. Further inquiries can be directed to the corresponding author.
